# Pituitary metastases: presentation and outcomes from a pituitary center over the last decade

**DOI:** 10.1007/s11102-020-01034-2

**Published:** 2020-03-18

**Authors:** K. Lithgow, I. Siqueira, L. Senthil, H. S. Chew, S. V. Chavda, J. Ayuk, A. Toogood, N. Gittoes, T. Matthews, R. Batra, S. Meade, P. Sanghera, N. Khan, S. Ahmed, A. Paluzzi, G. Tsermoulas, N. Karavitaki

**Affiliations:** 1 Institute of Metabolism and Systems Research, College of Medical and Dental Sciences, University of Birmingham, Birmingham, UK; 2 Centre for Endocrinology, Diabetes and Metabolism, Birmingham Health Partners, Birmingham, UK; 3 Department of Endocrinology, Queen Elizabeth Hospital, University Hospitals Birmingham NHS Foundation Trust, Birmingham, UK; 4 Department of Radiology, Queen Elizabeth Hospital, University Hospitals Birmingham NHS Foundation Trust, Birmingham, UK; 5 Department of Ophthalmology, Queen Elizabeth Hospital, University Hospitals Birmingham NHS Foundation Trust, Birmingham, UK; 6 Department of Oncology, Queen Elizabeth Hospital, University Hospitals Birmingham NHS Foundation Trust, Birmingham, UK; 7 Department of Ear, Nose & Throat, Queen Elizabeth Hospital, University Hospitals Birmingham NHS Foundation Trust, Birmingham, UK; 8 Department of Neurosugery, Queen Elizabeth Hospital, University Hospitals Birmingham NHS Foundation Trust, Birmingham, UK

**Keywords:** Pituitary, Metastases, Metastatic, Malignancy, Cancer, Hypopituitarism, Diabetes insipidus

## Abstract

**Purpose:**

Highlight and characterize manifestations, diagnostic/management approaches and outcomes in a contemporary cohort of patients with pituitary metastases (PM) from a large European pituitary center—over 10 years.

**Methods:**

Retrospective review of PM cases between 1/2009 and 12/2018. Clinical, laboratory, imaging data at PM detection and during follow-up were analysed.

**Results:**

18 cases were identified (14 females; median age at diagnosis 61.5 years). Most common primary malignancies were lung (39%) and breast (32%). Most frequent presenting manifestation was visual dysfunction (50%). Gonadotrophin, ACTH, TSH deficiency were diagnosed in 85%, 67%, 46% of cases, respectively; diabetes insipidus (DI) was present in 17%. 33% of cases were detected during investigation for symptoms unrelated to PM. PM management included radiotherapy (44%), transsphenoidal surgery (17%), transsphenoidal surgery and radiotherapy (6%) or monitoring only (33%). One-year survival was 49% with median survival from PM detection 11 months (range 2–47).

**Conclusions:**

In our contemporary series, clinical presentation of PM has evolved; we found increased prevalence of anterior hypopituitarism, decreased rates of DI and longer survival compared with older literature. Increased availability of diagnostic imaging, improvements in screening and recognition of pituitary disease and longer survival of patients with metastatic cancer may be contributing factors.

## Introduction

Pituitary metastasis (PM) is a rare manifestation of malignancy [1–5]. The reported prevalence varies depending on the method of assessment; 0.4% in radiological [6], 1% in surgical [1, 7], and between 0.14 and 28.1% in autopsy series [1]. Proposed mechanisms of PM include direct hematogenous spread, spread via the hypothalamic-portal vessels, meningeal spread via the suprasellar cistern, or extension from juxtasellar or skull base metastases [1]. The posterior pituitary has been considered to be particularly susceptible to metastatic spread, mainly due to receiving blood supply from the systemic circulation, in contrast to the anterior gland which is supplied through the hypophyseal portal system [8].

Clinical presentation is variable and includes visual field impairment or optic neuropathy, cranial nerve palsies, anterior pituitary dysfunction or diabetes insipidus (DI) [1–5, 9]. In earlier series, DI was a prominent presenting manifestation pointing towards the diagnosis of PM in a patient with known malignancy [1]. PM is most frequently detected in patients with a history of cancer, with breast and lung being the most common primary sites [1–5, 9]; it may also be the first manifestation of malignancy or detected incidentally [1, 3, 4, 6]. Imaging findings can be non-specific and are often not helpful in discriminating between PM and pituitary adenoma or other sellar lesion; however, rapid growth and invasion of parasellar structures should raise suspicion of pituitary metastatic infiltration [1, 3]. Management options include surgery and radiotherapy (alone or in combination), as well as, depending on the primary malignancy, chemotherapy, immunotherapy or hormonal therapy (such as tamoxifen for breast cancer). Evidence on the outcomes of these approaches is limited [7, 9–13], likely owing to the short survival of patients with PM.

Earlier diagnosis and appropriate management can potentially impact the outcome and quality of life of patients with PM. A significant proportion of the literature in this field (case reports and small case series) was published over a decade ago [1, 2]. Given advances in the sensitivity and availability of diagnostic imaging, as well as in the treatments and survival of cancer patients, it is anticipated that the presentation, natural history and prognosis of PM will have evolved, necessitating up to date information on contemporary clinical practice. Therefore, with this study, we aimed to elucidate the presenting manifestations, diagnostic and management approaches, as well as the outcomes of patients with PM from a large Pituitary center in Europe over the last 10 years.

## Patients and methods

Records of all cases with the diagnosis of PM included in the Pituitary Registry of the Department of Endocrinology, Queen Elizabeth Hospital Birmingham, UK between 1/2009 and 12/2018 were reviewed. Eligible cases had either histopathological confirmation of PM, or were diagnosed based on clinical and radiological features (history of underlying malignancy and imaging confirming the presence of a sellar mass with features, according to expert neuroradiology review, suggestive of metastatic infiltration or with rapid increase in its size). Clinical, laboratory and imaging data, as well as management approaches and outcomes were collected. Imaging characterization of the PM relied on brain and pituitary MRIs (pre- and post-contrast, T1- and T2-weighted images). Management approach for each PM case was individualized based on the decisions of the multidisciplinary oncology, neurosurgical and endocrine team.

Gonadotrophin deficiency was diagnosed based on low or inappropriately normal gonadotrophins with serum testosterone below the reference range in men, low or inappropriately normal gonadotrophins with low serum estradiol in premenopausal women, and gonadotrophins below the reference range for age in post-menopausal women. TSH deficiency was defined based on free T4 below the reference range in the presence of a low or inappropriately normal TSH value. ACTH deficiency was defined based on a 0900 h serum cortisol < 100 nmol/L in the absence of exogenous corticosteroid therapy, or an inadequate response to the 250 mcg short Synacthen test. DI was defined based on the presence of hypotonic polyuria with increased serum osmolality or serum sodium above the reference range and subsequent therapeutic response to desmopressin. Patients on medications potentially affecting pituitary hormone axes (e.g. opioids, glucocorticoids) were not included in the evaluation of the rates of pituitary hormone deficits attributed to PM.

The study was retrospective and involved no intervention beyond routine patient care. It was registered with and approved as an audit by the University Hospitals Birmingham NHS Foundation Trust.

### Statistical analysis

Percentages were calculated for categorical data and medians with ranges for continuous variables. Tumor progression-free curves and overall survival curves were generated by the Kaplan–Meier method. Statistical analyses were performed by IBM SPSS Statistics for Windows, Version 22.0. Armonk, NY: IBM Corp.

## Results

Eighteen cases were identified [14 females and 4 males with median age at PM detection 61.5 years (range 44–75)]. Their clinical characteristics are shown in Table 1. In all patients who underwent biopsy or surgical resection, the diagnosis of PM was confirmed by positive pathology (n = 8); in the remaining ones, diagnosis was based on clinical/radiological features.

**Table 1:** Clinical features of patients at the time of detection of pituitary metastases, management and outcomes

Case	Age	Sex	Site of Primary Malignancy	Clinical manifestations	Anterior pituitary hormone deficiencies	DI	Other sites of metastases	Management	Outcome	Time to growth (months)
1	65	F	Lung	Visual dysfunction (VF defects and CN III palsy)	FSH/LH-Yes TSH-Yes ACTH-Yes	No	Yes^a^: liver, brain (cerebral), spine	Radiotherapy (whole brain)	Regressed	N/A
2	53	F	Breast	Symptoms/signs of cortisol deficiency and VF defects	FSH/LH-Yes TSH -Yes ACTH-Yes	No	No	Surgery and adjuvant radiotherapy (whole brain)	Regressed	N/A
3	67	M	Prostate	Visual dysfunction (reduction in VA and VF defects)	FSH/LH-Yes TSH-No ACTH-No	No	No	Radiotherapy	Unknown	N/A
4	61	M	Lung	DI	No information	Yes	No	Monitoring	Growth	3
5	63	F	Lung	Visual dysfunction (VF defects)	FSH/LH-Yes TSH-Yes ACTH-Yes	No	Yes^a^: adrenal glands, mediastinal lymph nodes	Surgery	Growth	4
6	50	F	Breast	Visual dysfunction (VF defects)	FSH/LH-Yes TSH-No ACTH-no information	Yes	Yes: lymph nodes, bone, liver	Radiotherapy	Growth	9
7	64	F	Melanoma	Visual dysfunction (CN VI palsy)	None	No	Yes: lung, liver, bone, mesentery	Monitoring	Unknown	N/A
8	55	F	Breast	Visual dysfunction (rapid visual loss leading to blindness)	FSH/LH-Yes TSH-Yes ACTH -Yes	No	No	Surgery	Unknown	N/A
9	62	F	Lung	Symptoms unrelated to PM (abnormal sensation in R side of face, change in function of left hand)	No information	No	Yes^a^: brain (thalamus)	Radiotherapy	Unknown	N/A
10	56	F	Melanoma	Visual dysfunction (VF defects)	FSH/LH-Yes TSH-Yes ACTH-Yes	No	No	Surgery	Growth	45
11	74	F	Breast	Symptoms/signs of cortisol deficiency	FSH/LH-Yes TSH-no information ACTH-Yes	Yes	Yes: lung, skin, bone	Monitoring	Unknown	N/A
12	75	M	Renal	Visual dysfunction (CN III and VI palsies and VF dysfunction)	FSH/LH-Yes TSH-No ACTH-Yes	No	Yes: lung, pancreas	Monitoring	Growth	3
13	57	F	Lung	Symptoms unrelated to PM (ataxia and vomiting)	No information	No	Yes^a^: adrenal, bone, brain (cerebellum)	Radiotherapy (whole brain)	Unknown	N/A
14	73	F	Lung	Symptoms unrelated to PM (unsteady gait and L sided facial droop)	FSH/LH-Yes TSH-No ACTH-No	No	Yes: brain (cerebral)	Monitoring	Growth	15
15	66	M	Prostate	Visual dysfunction (CN III palsy)	FSH/LH-no information TSH-No ACTH-no information	No	Yes: bone, lymph nodes	Radiotherapy (whole brain)	Regressed	N/A
16	59	F	Lung	Incidental finding during staging imaging	No information	No	Yes^a^: brain (cerebral)	Radiotherapy (whole brain)	Unknown	N/A
17	44	F	Breast	Symptoms unrelated to PM (seizures)	FSH/LH-No TSH-No ACTH-No	No	Yes: bone, brain (cerebral), liver, spleen	Monitoring	Growth	23
18	55	F	Breast	Incidental finding during follow-up imaging for a co-existent tentorial meningioma	FSH/LH-Yes TSH-Yes ACTH-Yes	No	Yes: bone	Radiotherapy	Unknown	N/A

*VF* visual field, *VA* visual acuity, *FSH* follicle stimulating hormone, *LH* luteinizing hormone, *ACTH* adrenocorticotropic hormone, *DI* diabetes insipidus, *N/A* not applicable

^a^Indicates PM was first manifestation of metastatic disease

Primary malignancy was lung cancer (39%, n = 7), breast cancer (33%, n = 6), prostate cancer (11%, n = 2), melanoma (11%, n = 2) and renal cell carcinoma (6%, n = 1). In 72% (n = 13) of the cases, there was a known history of malignancy [median time from diagnosis of primary tumor to PM detection 29 months (range 10–244)]. In the remaining 28% (n = 5), PM was the first manifestation leading to diagnosis of the primary cancer; this group had presented with cranial nerve palsy (n = 1), visual fields deficit (n = 1), neurological symptoms related to synchronous metastatic deposit elsewhere in the brain (n = 2) and rapid growth of a pituitary mass during follow-up of a presumed macroadenoma (n = 1). Other metastases at the time of PM detection were known in 72% (n = 13) of patients. Amongst the breast cancers, human epidermal growth factor receptor 2 (HER2) was overexpressed in 60% (n = 3/5) and 100% (n = 5/5) were estrogen receptor positive.

Visual dysfunction was the most common presentation of PM [50%, n = 9; disturbances of visual fields and/or visual acuity (n = 5) or cranial nerve palsies (n = 4)]. Clinical features of hypopituitarism were described in 17% (n = 3); two patients had symptoms of cortisol deficiency and one had manifestations of DI. In 33% (n = 6), PM was detected during investigations for reasons unrelated to pituitary disease [neurological symptoms secondary to other brain metastases (n = 4), staging of the primary cancer (n = 1) and to re-evaluate a previously known meningioma (n = 1)]. Gonadotrophin deficiency was diagnosed in 85% (11/13), ACTH deficiency in 67% (8/12), TSH deficiency in 46% (6/13) and DI was found in 17% (3/18) of the patients. Formal visual review revealed optic nerve dysfunction (visual field and/or visual acuity compromise) in 57% (8/14) and cranial nerve palsies in 22% (4/18) of the cases. On pituitary MRI, suprasellar extension was seen in 94% (17/18), cavernous sinus extension in 39% (7/18), sphenoid sinus invasion in 11% (2/18) and extension through the sellar floor to the clivus in 22% (4/18) of the patients.

Management decisions were individualised and were based on the outcome of multidisciplinary team meetings; the main indication for surgery was visual deterioration and this option was adopted whenever possible. Primary management for PM was fractionated radiotherapy in 44% (n = 8), transsphenoidal surgery in 17% (n = 3), transsphenoidal surgery with adjuvant fractionated radiotherapy in 6% (n = 1) and monitoring only in 33% (n = 6). Follow-up imaging was available for ten cases [median monitoring 9 months (range 2–45)]. In the remaining eight, this was not arranged due to limited life expectancy (n = 6, all died within 12 months of diagnosis) or because follow-up of PM was done with neuro-ophthalmology assessment (n = 2, one case died 37 months after diagnosis and the second remained alive at time of last review 3 months after PM diagnosis). Of the ten cases with follow-up imaging (managed by radiotherapy n = 3, surgery n = 2, surgery and radiotherapy n = 1, monitoring n = 4), radiological progression was seen in seven at a median time of 9 months (range 3–45) since primary PM management. Four of these patients [previously managed by transsphenoidal surgery (n = 1) or monitoring (n = 3)] required intervention due to threat to vision [transsphenoidal surgery with adjuvant fractionated radiotherapy (n = 2), fractionated radiotherapy (n = 1), or transsphenoidal surgery (n = 1)]. A second episode of PM radiological progression was detected in one patient 10 months after pituitary surgery and radiotherapy and was managed by another course of external irradiation.

At last follow-up, gonadotrophin deficiency was present in 92% (12/13), ACTH deficiency in 82% (9/11), TSH deficiency in 64% (9/14) and DI in 22% (4/18). Patients were on glucocorticoid, levothyroxine, and desmopressin replacement as appropriate. Gonadal hormone replacement was not offered in any of the cases. Eight of the 11 cases with baseline optic nerve dysfunction had follow-up visual assessment; vision was improved in four, stable in two and deteriorated in two cases.

**Fig. 1:**
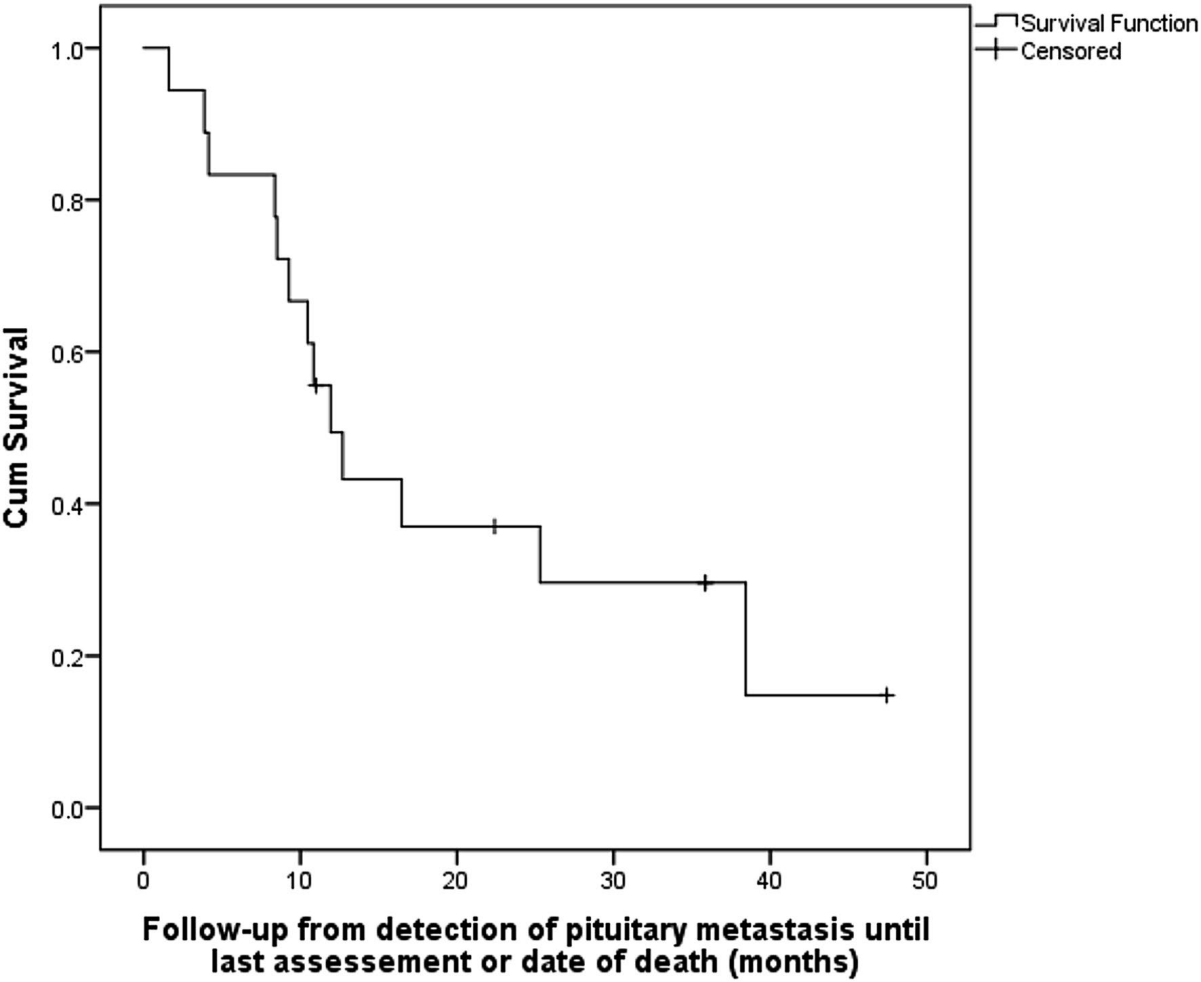
Survival of the patients since the diagnosis of pituitary metastasis

Median clinical follow-up (from imaging detecting PM until last assessment or death) was 11 months (range 2–47). During this period, 13 patients died as a result of their malignancy at a median time from PM detection 11 months (range 1–37) [median age at death 63 years (range 52–74)]. The probability of survival was 49% and 37% at 1 and 2 years, respectively (Fig. 1).

## Discussion

In this series of patients presenting with PM over the last decade in our pituitary centre, we reviewed their clinical characteristics and outcomes. Compromised vision was the most common presenting manifestation, whereas DI was less frequent than described in earlier studies. In around one third of the cases, PM was detected during investigations for symptoms not related to pituitary disease. Importantly, survival was 49% at 1 year, reflecting advances in the diagnosis and management of malignant disease.

In agreement with previous literature [1, 4, 5, 9], we found that lung and breast were the predominant primary sites of malignant disease; nonetheless, pituitary metastatic infiltration can be seen in almost any type of malignancy [1, 4, 5, 9]. In a recently published study, breast cancers overexpressing HER2 seemed to be more prone to metastasise to the pituitary gland [9]. In our breast cancer cases, HER2 was overexpressed in 60%.

Compromised vision was the most common presenting manifestation of PM found in 50% of our cases and further formal visual review revealed optic nerve dysfunction in 57%. He et al. [2] and Schill et al. [9] reported rates visual loss of 41% and 42%, respectively, as opposed to 27% from a review of cases published in 2004. Increased availability and improvements in neuro-ophthalmological assessment allowing detection of more subtle visual disturbances could be a potential explanation for this rise. Anterior hypopituitarism was diagnosed in a significant number of our cases. Despite this, symptomatology of hypopituitarism was the presenting manifestation in a much lower rate of patients. The possibility that the symptoms and signs of hypopituitarism were attributed to the underlying malignancy and, at least initially, were not associated with PM cannot be excluded. Furthermore, DI was present in only 17% of the cases suggesting that the clinical presentation of PM has evolved. In earlier literature, posterior lobe dysfunction was more common than anterior hypopituitarism [8], and DI was the most frequent presenting manifestation of PM, even reported at 100% in some series [1]. Our findings and other recent publications contradict this. He et al. [2] in a systematic review and pooled analysis of 248 PM cases, compared clinical features of 190 patients previously reviewed by Komninos et al. [1] (published 1970–2002) with 58 cases reported from 2004 to 2011. Their analysis demonstrated that the later 58 cases had significantly lower rates of DI and higher rates of visual dysfunction, cranial nerve palsies and anterior pituitary dysfunction [2]. Furthermore, in a recently published nation-wide series of 38 cases of PM, majority of which presented between 2011 and 2013, rates of DI were 26%, of gonadotrophin deficiency 88%, of TSH deficiency 65%, and for ACTH deficiency 71% [9]. Factors explaining this change may be the earlier detection of PMs, increased awareness of manifestations of hypopituitarism, as well as better availability and wider use of relevant biochemical testing. Furthermore, improvements in cancer screening and diagnosis in general may also contribute to early detection of PM.

PM was the first manifestation of malignancy in 28% of our patients. This rate varies in the literature between 25 and 64% [1, 3, 4, 14] and may be influenced by the differences in the primary malignancies included in various series. Schill et al. reported that 63% of PM from lung cancers were detected before or within the first year after diagnosis of the primary tumour, whereas 53% of PM from breast cancers were detected 10 years or more after diagnosis of the primary tumour [9]. Notably, in our series, all cases where PM was the first manifestation of malignancy had a lung primary. Time of publication may also be a contributing factor; the highest proportions of PM as the first manifestation of malignancy were seen in older series [1, 14], potentially reflecting improved screening and earlier diagnosis of common primary malignancies in recent decades. In 33% of our cases, PM was detected during investigations for symptoms not related to pituitary disease. The prevalence of PM which are latent at detection has not been previously well-characterised. However, the pooled prevalence of PM is higher in autopsy series compared with that of clinical or surgical ones [1, 2], suggesting that many cancer patients have latent PM which may be detected incidentally.

Differential diagnosis of PM from other sellar lesions is often difficult and poses challenges, especially when there is no history of primary malignancy. Signal characteristics on MRI are variable and include hypointensity on T1 weighted imaging, hypo- or hyperintensity on T2 weighted imaging, and post-contrast enhancement [1, 4, 9]. Extension, particularly suprasellar, is common [9], and a “dumbbell” shape on sagittal view is frequently seen [1, 4, 9]. However, extension is not always present; some PMs present as a sellar mass or with thickening of the pituitary stalk [3]. Other imaging features include infiltration into adjacent tissues and rapid increase in size [1, 4]. Increased uptake on ^18^F-fluorodeoxyglucose (FDG) positron emission tomography-computed tomography (PET/CT) has been observed in PM, however this is a non-specific finding as it can also be observed in pituitary adenomas, hypophysitis, Langerhans cell histiocytosis or indeed normal pituitary tissue [15].

The management of PM which also depends on the extent of systemic disease, aims to provide symptomatic relief (mainly of visual disturbances) with a favourable impact on quality of life and to prevent further enlargement of the lesion. In our series, visual disturbances improved or remained stable in 75% of the patients with available follow-up data. Visual outcomes following treatment have been reported in a limited number of studies; improvements in vision and cranial nerve dysfunction have been described in 30–100% and 50–100%, respectively after surgery [7, 12, 13]. Stereotactic radiosurgery (SRS) has been shown to be effective for controlling growth of PM [10, 11, 16] and improvement in cavernous sinus cranial nerve palsies has been observed following SRS [10, 11, 16]. Hypofractionated SRS has shown promise for pituitary adenomas in achieving tumour control with low toxicity to the optic pathway [17], though experience with this strategy for PM is thus far limited to a small case series [18]. Progression of PM depends on the type and stage of primary malignancy, management strategies and duration of follow-up and relevant data are scarce [7, 9–13]. In our series, 7 out of 10 patients with available imaging showed PM progression at a median time of 9 months since primary PM management, which in 57% of cases included monitoring.

Survival after the diagnosis of PM depends on the type and stage of primary cancer. Historically, PM had been associated with aggressive disease, end stage malignancy, and reduced life expectancy [1] with earlier series reporting mean survival of 6–7 months [1]. Ntyonga-Pono et al. [19] in a group of patients presenting prior to 1999 reported that only 10% lived beyond 1 year. However, survival after PM detection appears to be improving over time, possibly due to earlier diagnosis of pituitary metastatic infiltration and to improved life expectancy of cancer patients. In our series, survival was 49% and 37% at 1 and 2 years, respectively. This rate may be also be influenced by referral bias as in our tertiary center more advanced cancer cases are likely to be referred. In agreement with our data, Schill et al. [9] demonstrated 1 year survival of 50% in a series of patients diagnosed with PM between 1996 and 2018. Recognition that a large proportion of patients with PM may survive beyond 1 year is clinically significant, as it will facilitate decisions on interventions aimed at restoring vision or preventing visual loss, and improving quality of life.

Limitations of our study include its small size and retrospective design (although prospective studies for this rare condition may not be a realistic goal) and the lack of pathological confirmation of PM in a number of cases. Nonetheless, the rapid mass enlargement and the imaging appearances not consistent with a benign mass were in favour of metastatic disease. Advantages include its focus on contemporary practice of the last 10 years from a large pituitary centre in Europe and the reasonable follow-up duration after PM detection allowing survival analysis.

In our single centre cohort of PMs, we illustrate the presentation and outcomes of PM in the era of increasing survival of cancer patients and wide availability of diagnostic imaging. We found increased prevalence of anterior hypopituitarism and decreased rates of DI possibly also related with improvements in screening for pituitary dysfunction. However, 33% of our cases were detected during investigations for symptoms not related with pituitary disease suggesting that clinicians should be alert for screening for hypopituitarism in patients with known malignancy, particularly in breast or lung. The improvement in survival is encouraging and can guide decisions on decisions for active therapeutic interventions for the PM that will provide benefit to the patients (e.g. resolution of visual disturbances with favourable impact on quality of life). Further outcome-oriented studies are needed to determine the safety and efficacy of different management strategies for PM.
